# IDO and CD40 May Be Key Molecules for Immunomodulatory Capacity of the Primed Tonsil-Derived Mesenchymal Stem Cells

**DOI:** 10.3390/ijms22115772

**Published:** 2021-05-28

**Authors:** Hyun-Joo Lee, Harry Jung, Dong-Kyu Kim

**Affiliations:** 1 Institute of New Frontier Research Team, Hallym University College of Medicine, Chuncheon 24253, Korea; leekul79@gmail.com (H.-J.L.); harry880219@gmail.com (H.J.); 2 Department of Otorhinolaryngology-Head and Neck Surgery, Chuncheon Sacred Heart Hospital, Hallym University College of Medicine, Chuncheon 24253, Korea

**Keywords:** tonsil, mesenchymal stem cell, indoleamine 2,3-dioxygenase, CD40

## Abstract

**Background:** Tonsil-derived mesenchymal stem cells (T-MSCs) were reported to have suppressive effect on T cells, yet much remains unknown about the underlying mechanisms supporting this effect. We investigated the underlying mechanism of the immunomodulatory effect of T-MSCs on immune cell proliferation and cytokine production. **Methods:** We isolated T-MSCs from human palatine tonsil and evaluated the immunomodulatory capacity using RT-PCR, ELISA, and flow cytometry. Additionally, we assessed the expression of various soluble factors and several costimulatory molecules to detect the priming effect on T-MSCs. **Results:** T-MSCs significantly inhibited the immune cell proliferation and cytokine expression (TNF-α and IFN-γ) in the direct co-culture, but there was no suppressive effect in indirect co-culture. Additionally, we detected a remarkably higher expression of indoleamine 2,3-dioxygenase (IDO) in the primed T-MSCs having co-expression CD40. Moreover, immune cells or CD4^+^ T cells showed lower TNF-α, IFN-γ, and IL-4 expression when the primed T-MSC were added; whereas those findings were reversed when the inhibitor for IDO (not IL-4) or CD40 were added. Furthermore, T-bet and GATA3 levels were significantly decreased in the co-cultures of the primed T-MSCs and CD4^+^ T cells; whereas those findings were reversed when we added the neutralizing anti-CD40 antibody. **Conclusions:** Primed T-MSCs expressing IDO and CD40 may have immunomodulatory capacity via Th1-mediated and Th2-mediated immune response.

## 1. Introduction

Mesenchymal stem cells (MSCs), which are capable of differentiating into various cell types, are an attractive cell source for the repair and regeneration of damaged tissues [1,2]. Additionally, MSCs have regulatory effects on immune responses by suppressing T cell proliferation stimulated by allogeneic lymphocytes, dendritic cells, and phytohemagglutinin (PHA) [3,4,5]. Such immunosuppressive activity may occur through cell contact or soluble factors such as interferon-gamma (IFN-γ) and indoleamine 2,3-dioxygenase (IDO) [4,6,7,8]. However, although bone marrow (BM) and adipose tissue have been the major stem cell source for the isolation of multipotent MSC, there are critical limitations. The harvesting of BM-derived MSC is a highly invasive technique and the differentiation potential of adipose tissue-derived MSC usually shows a significant decline with the increase of the donor’s age [9,10].

Tonsil-derived mesenchymal stem cells (T-MSCs) have recently been introduced and have been getting a great deal of attention in various medical fields due to them being easier to harvest than BM-MSCs. They also have more immunomodulatory effects than adipose-derived MSCs [11,12,13,14]. Some studies showed that exogenously administered T-MSCs migrate to damaged tissue sites and participate in tissue repair by immune regulatory functions in a mouse model of liver injury [15,16]. Other studies have also demonstrated an immunomodulatory effect of T-MSCs in a mouse model of allergic rhinitis [14,17]. Moreover, several in vitro studies revealed that T-MSCs exert immunomodulatory effects on T lymphocyte proliferation [18,19,20]. However, the exact mechanism related to immunomodulatory effects of T-MSCs has not been fully characterized. Therefore, in this study, we investigated the underlying mechanism regarding immunomodulatory effects of T-MSCs on immune cell proliferation and cytokine production.

## 2. Results

### 2.1. Characterization of Tonsil Derived Mesenchymal Stem Cell

**Figure 1:**
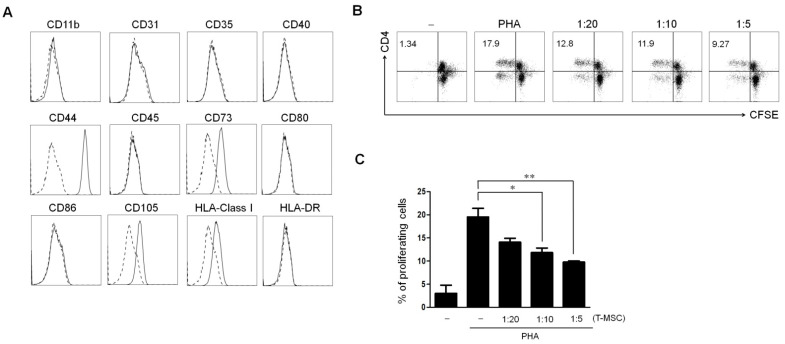
Characterization and immunosuppressive activity of tonsil-derived mesenchymal stem cells (T-MSCs). (**A**) T-MSCs are characterized for marker expression by flow cytometry. Dashed histograms indicate staining with isotype control antibodies and solid histograms denote the specific expression of each indicated marker. In vitro immunosuppressive activity of T-MSCs according to different concentrations (absence, 5 × 10^4^, 1 × 10^5^, and 2 × 10^5^) on (**B**) CD4^+^ T cell and (**C**), whole immune cells are determined by CFSE assay. Dot blots are representative of each independent experiment and the graphic data is the ± SEM from each independent experiment (* *p* < 0.05 and ** *p* < 0.001). T-MSC: tonsil-derived mesenchymal stem cell; PHA, phytohemagglutinin; CC, cell–cell contact; TW, transwell.

To characterize the profile of T-MSCs, we performed flow cytometry analyses (Figure 1A). We found that cells were positively labeled with human MSCs markers, such as CD44, CD73, CD105, and HLA-Class I, whereas costimulatory molecules marker of CD40, CD80, and CD86 and hematopoietic/endothelial markers of CD11b, CD31, CD35, CD45, and HLA-DR were negatively expressed. To investigate the immunosuppressive effect of T-MSCs, we compared the proliferation of CD4^+^ T cells according to the concentration of T-MSCs (absence, 5 × 10^4^, 1 × 10^5^, and 2 × 10^5^) (Figure 1B). We stimulated immune cells with PHA, which acts as an activator of leukocyte activation. After stimulation, we found that T-MSCs suppressed the proliferation of CD4^+^ T cells in a dose-dependent manner. Additionally, we detected decreasing proliferation of whole immune cells on the addition of T-MSC in a dose-dependent manner (Figure 1C).

### 2.2. Comparison of Immunosuppressive Function between Direct (Cell–Cell Contact) and Indirect (Transwell) Co-Culture

**Figure 2:**
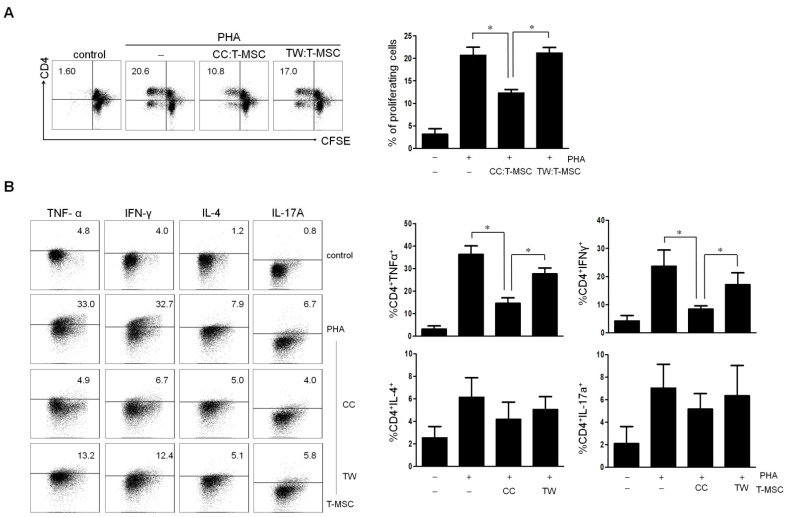
Assessment of tonsil-derived mesenchymal stem cells (T-MSCs)-mediated immunosuppressive function. (**A**) Flow cytometric analysis of the proliferating immune cells after direct or indirect co-cultured with T-MSCs for 4 days. (**B**) Flow cytometric analysis of TNF-α, IFN-γ, IL-17A, and IL-4 expression in CD4^+^ T cells after direct or indirect co-cultured with T-MSCs for 4 days. Dot blots are representative of each independent experiment and the graphic data is the ± SEM from three independent experiments (* *p* < 0.05). T-MSC: tonsil-derived mesenchymal stem cell; PHA, phytohemagglutinin; CC, cell–cell contact; TW, transwell.

Cell–cell contact between MSCs and T cells is known to be essential for MSC-mediated T cell regulation [6,21,22]. We investigated whether the proliferation and cytokine expression of the immunosuppressive potential of T-MSCs could be different by direct co-culture and indirect transwell culture. In direct co-cultures, T-MSCs (1 × 10^5^ cells/well) were co-cultured with immune cells at a ratio of 1:10, whereas in transwell cultures, T-MSCs were seeded in the upper chamber and immune cells were seeded in the lower chamber. After T-MSCs were co-cultured with immune cells for 4 days, we detected that T-MSCs significantly inhibited the proliferation of the immune cells in the direct co-culture, but there was no suppressive effect in the indirect co-culture (Figure 2A). Additionally, TNF-α and IFN-γ expression were significantly lower in CD4 T cells from direct co-culture than those from indirect co-culture. However, IL-4 and IL-17A expression have been shown moderate reduction by direct contact with T-MSCs and there was also no significant difference between direct and indirect co-cultures (Figure 2B).

### 2.3. Effect of Priming on Tonsil Derived Mesenchymal Stem Cell

**Figure 3:**
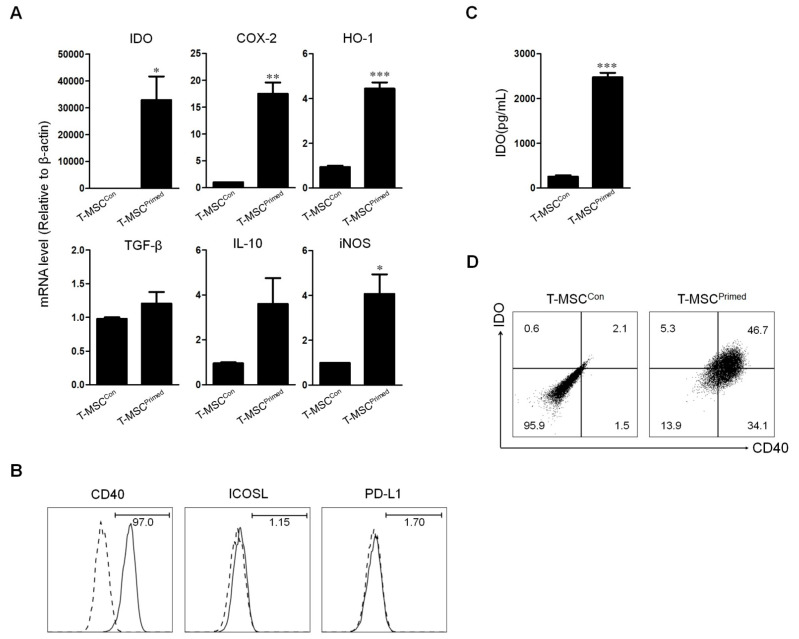
Effect of priming on the production of critical immunomodulatory factors in tonsil-derived mesenchymal stem cell (T-MSC). (**A**) Comparison of soluble factors expression, including IDO, COX-2, HO-1, TGF-β1, IL-10, and iNOS between T-MSC^Con^ and T-MSC^Primed^ using RT-PCR (* *p* < 0.05, ** *p* < 0.001, *** *p* = 0.0008). (**B**) Comparison of costimulatory molecule expression, such as PD-L1, ICOSL, and iNOS between T-MSC^Con^ and T-MSC^Primed^ using flow cytometry. (**C**) IDO inductivity of T-MSC^Primed^ is confirmed by ELISA (*** *p* = 0.0004). (**D**) Protein expression of CD40 and IDO is confirmed by flow cytometry between T-MSC^Con^ and T-MSC^Primed^. T-MSC^Con^, tonsil-derived mesenchymal stem cell with the addition of vehicle control; T-MSC^Primed^, tonsil-derived mesenchymal stem cell primed with TNF-α and IFN-γ; IDO, indoleamine 2,3-dioxygenase quantification; HO-1, heme oxygenase; PD-L1, programmed death-ligand 1; ICOSL, inducible T cell costimulator ligand.

Several studies have reported higher immunosuppressive effects in T-MSCs with inflammatory stimuli [23,24,25]. Thus, to verify whether the priming with TNF-α and IFN-γ could strengthen the inhibitory function of T-MSCs, we evaluated the expression of soluble factors and costimulatory molecules related to immunosuppressive function. We demonstrated that the expression of IDO, COX-2, HO-1, TGF-β1, IL-10, and iNOS mRNA levels were significantly increased in the T-MSC^Primed^ compared to the T-MSC^Con^ without priming treat (Figure 3A). Using flow cytometry, we also found a higher expression of CD40 in the T-MSC^Primed^ than in the T-MSC^Con^, but there was no significant difference between PD-L1 and ICOSL expression (Figure 3B). Moreover, we detected a considerable increased IDO mRNA expression in the T-MSC^Primed^ (Figure 3C). Additionally, we detected the T-MSC^Primed^ having a CD40 positive population producing IDO (Figure 3D).

### 2.4. Effect of IDO and CD40 on T Cell Proliferation and Cytokine Expression

**Figure 4:**
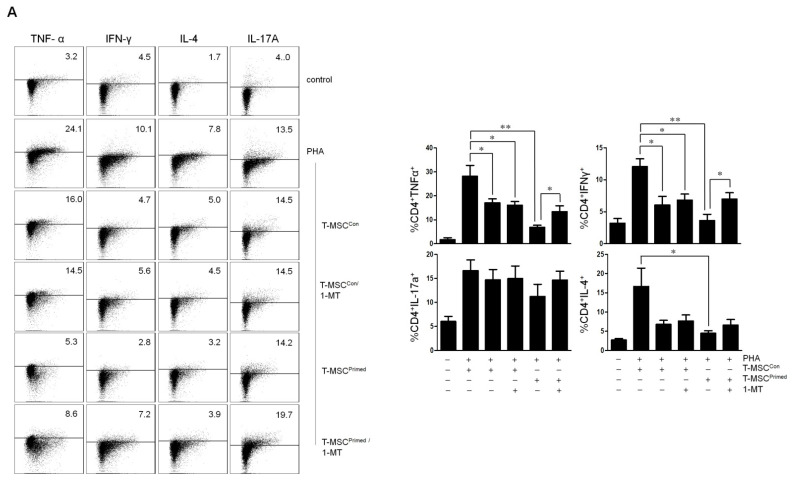

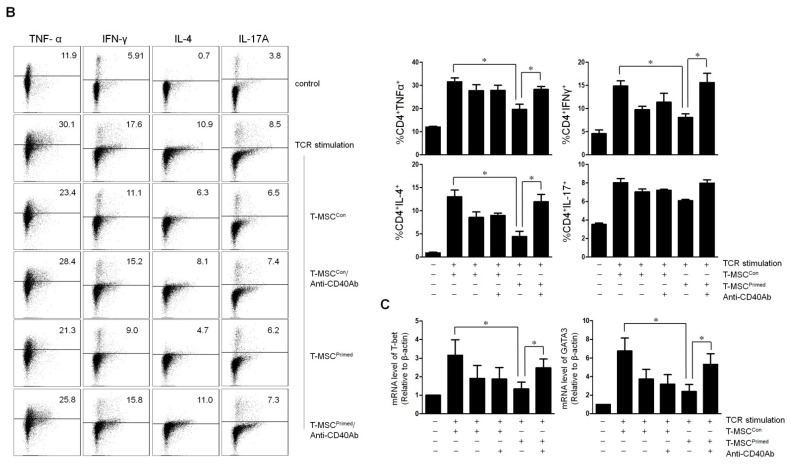
Indoleamine 2,3-dioxygenase quantification (IDO) and CD40 are key molecules on the immunomodulatory effect of the primed tonsil-derived mesenchymal stem cells (T-MSCs). (**A**) Expression of TNF-α, IFN-γ, and IL-4 in CD4^+^ T cells according to the different groups (control, PHA, T-MSC^Con^, T-MSC^Primed^ and inhibitor IDO) (**B**) Expression of TNF-α, IFN-γ, and IL-4 in CD4^+^ T cells according to the different groups (control, TCR, T-MSC^Con^, T-MSC^Primed^, and Anti-CD40Ab) (**C**) Expression level of transcription factors of Th1 (T-bet) and Th2 (GATA3) both in the co-cultures of T-MSCs and CD4^+^ T cells according to the different groups (control, TCR, T-MSC^Con^, T-MSC^Primed^, and Anti-CD40Ab). Dot blots are representative of each independent experiment. The graphic data is the ± SEM from each independent experiment. * *p* < 0.05 and ** *p* < 0.01. T-MSC^Con^, tonsil-derived mesenchymal stem cell with the addition of vehicle control; T-MSC^Primed^, tonsil-derived mesenchymal stem cell primed with TNF-α and IFN-γ; Anti-CD40Ab, Neutralizing anti-CD40 antibodies.

Next, we explored whether the expression of IDO in T-MSC^Primed^ affects the inhibition of cytokine expression in activated immune cells. To assess the inhibitory effect, we co-cultured T-MSCs and CD4^+^ T cells stimulated with PHA. We observed that the T-MSC^Primed^ showed lower expression of TNF-α, IFN-γ, and IL-4 in CD4^+^ T cells than in T-MSC^veh^. Moreover, the T-MSC^Primed^ with the addition of 1-MT (inhibitor for IDO) in CD4^+^ T cells showed upregulated TNF-α and IFN-γ expression (Figure 4A).

Additionally, to investigate the immunosuppressive properties of CD40 in the T-MSC^Primed^, CD4^+^ T cells stimulated with anti-CD3 and anti-CD28 antibodies were co-cultured with T-MSC^Primed^ or T-MSC^Con^. Flow cytometry showed that T-MSC^Primed^ significantly suppressed TNF-α, IFN-γ, and IL-4 production of CD4^+^ T cells, whereas the addition of neutralizing anti-CD40 antibody reversed the inhibition of TNF-α, IFN-γ, and IL-4 production (Figure 4B). Moreover, we observed that the expression of T-bet and GATA3 mRNA levels were significantly downregulated in the co-cultures of T-MSC^Primed^ and CD4^+^ T cells. However, T-bet and GATA3 expression was rescued when we added the neutralizing anti-CD40 antibody in the co-cultures of T-MSC^Primed^ and CD4^+^ T cells (Figure 4C).

## 3. Discussion

To date, there are few studies that have investigated the underlying mechanism of the immunomodulatory effect of T-MSCs on immune cell proliferation and cytokine production. In this study, we assessed the underlying mechanism related to the immunosuppressive capacity of T-MSCs. Using vitro analysis, we found that T-MSCs effectively mediate immunosuppressive function of immune cell proliferation and cytokine production in a cell–cell contact manner. Additionally, when T-MSCs were pretreated with TNF-α and IFN-γ, the inhibitory function of T-MSCs was enhanced with a higher expression of IDO and CD40. Moreover, we detected that T-MSC^Primed^ decreased the proliferation of immune cells. The production of TNF-α, IFN-γ, and IL-4 were also significantly downregulated. During the analysis of confirming the immunosuppressive properties of IDO and CD40 in the T-MSC^Primed^, we observed that the 1-MT rescued T-MSC^Primed^-mediated T cell proliferation and the production of cytokine expression, such as TNF-α and IFN-γ. Moreover, CD40 neutralization reversed the inhibition of T-MSC^Primed^-mediated TNF-α, IFN-γ, and IL-4 production.

T-MSCs have recently been introduced as a new source of MSC because they can easily be isolated from palatine tonsil tissues obtained from routine tonsillectomy procedures. The immunosuppression by the cell-to-cell contact between MSCs and T cells is well documented [6,21,22]. Consistent with these findings, we detected that T-MSCs significantly inhibited the proliferation of immune cell and cytokine expression (TNF-α/IFN-γ) in a cell-to-cell contact assay rather than a transwell assay. Previously, one study demonstrated that co-culture with T-MSCs after inducing Th2 substantially increased IL-4 and decreased IFN-γ. However, our study showed that T-MSCs significantly decreased IFN-γ but the trend towards decreased IL-4, because we used T-MSCs without inducing Th2. Additionally, we found that the immunosuppressive capacity of T-MSCs was enhanced by pretreatment with 10 ng/mL TNF-α and 20 ng/mL IFN-γ. The mRNA expression of IDO, COX-2, HO-1, and iNOS was significantly increased in the T-MSC^Primed^ compared to the T-MSC^Con^. Interestingly, among those soluble factors, we detected a remarkably increased expression of IDO mRNA. Previous studies have demonstrated an indispensable role of IDO in the immunomodulatory capacity of human MSCs [26,27,28]. This study also showed a higher concentration of IDO in T-MSC^Primed^ than T-MSC^Con^. Similar to our findings, one study demonstrated that the IFN-γ priming of amnion-derived MSCs induces high production of IDO [29]. Another study also showed increased IDO expression when MSCs are primed with inflammatory cytokines [30].

CD40 is a costimulatory protein found on antigen-presenting cells. The coupling of the CD40 ligand on T cells with CD40 on antigen-presenting cells can result in the activation of the antigen-presenting cells [31]. Additionally, the expression of the immune-stimulatory receptor, CD40 on myeloid-derived suppressor cells is required to induce T-cell tolerance and Treg accumulation [32]. However, apart from the production of regulatory metabolic enzymes, the repertoire of co-stimulatory and/or co-inhibitory molecules harnessing T-MSCs for immune regulation, including the role of CD40, is largely unknown. A recent study demonstrated that CD40 is upregulated on MSCs under the same conditions previously reported to induce IDO [33]. In this study, we also found that T-MSC^Primed^ had a higher concentration of CD40 compared to T-MSC^Con^. Moreover, T-MSC^Primed^ exhibited a phenotype co-expressing CD40 and IDO. Thus, these results imply that CD40 expressed with IDO may be significant to the immune capacity of T-MSCs.

In the functional study of CD40 in T-MSCs, to exclude the expression of CD40 in antigen-presenting cells, we isolated CD4 T cells from immune cells and then, purified CD4 T cells were co-cultured with T-MSCs under TCR stimulation. Unlike the action of T-MSCs on PHA stimulated immune cells, T-MSC^Con^ had no inhibitory function. Cytokine production of TCR stimulated CD4 T cells were suppressed only with T-MSC^Primed^. These results have shown that T-MSCs might be more effective in inhibiting the activation of CD4 T cells via regulation of antigen-presenting cells. Additionally, to confirm IDO and CD40 involvement in the process of immunosuppression, 1-MT and anti-CD40 antibodies were added to the co-cultures of T-MSC^Primed^ and CD4^+^ T cells. This reversed the inhibition of TNF-α, IFN-γ, or IL-4 production. Furthermore, the co-cultures of T-MSC^Primed^ and CD4^+^ T cells significantly inhibited the expression of T-bet and GATA-3, and those were rescued by treatment with CD40 neutralization. Collectively, these results indicate that T-MSC^Primed^ expressing IDO and CD40 may have immunomodulatory capacity via Th1-mediated and Th2-mediated immune responses. To the best of our knowledge, although only in vitro results were reported, this is the first study to explore the functional effect of CD40 and IDO co-expression in T-MSCs-mediated immunomodulation. However, in this study, we did not confirm the effect of CD40 and IDO expression of human T-MSCs using an in vivo study. Thus, it is not clear whether CD40 and IDO-coexpressing human T-MSCs have an immunosuppressive effect in vivo experiment. Therefore, we should perform an appropriate animal study to prove our concept.

In conclusion, T-MSCs may play an important role in immune-suppressive function via cell–cell contact. Additionally, T-MSC^Primed^ exhibited a phenotype co-expressing CD40 and IDO, which may play an important role in the immunomodulatory effect of T-MSC^Primed^ via Th1 and Th2 immune responses. Therefore, when T-MSCs are primed with inflammatory cytokines, their immunomodulatory effects may be used for clinical purposes.

## 4. Materials and Methods

This study was reviewed and approved by the Institutional Review Board of Hallym Medical University Chuncheon Sacred Hospital (Chuncheon, Korea, IRB No. 2016-04-041, 22-07-2016). Written informed consent was obtained from donors. This study adhered to the tenets of the Declaration of Helsinki.

### 4.1. Isolation and Characterization of Tonsil Derived Mesenchymal Stem Cell

In this study, tonsil tissues obtained from a pediatric tonsillectomy were cultured under stem cell-enrichment conditions in order to isolate T-MSCs. The isolated T-MSCs were characterized for several cell surface antigens by flow cytometry. The antibodies used for this analysis included anti-CD11b (Biolegend, San Diego, CA, USA), anti-CD31 (Biogems, Westlake Village, CA, USA), anti-CD35 (eBiosciences, San Diego, CA, USA), anti-CD40 (Biolegend), anti-CD44 (BD Biosciences, San Diego, CA, USA), anti-CD45 (Biogems), anti-CD73 (Biogems), anti-CD80 (Biolegend), anti-CD86 (Biolegend), anti-105 (BD Biosciences), anti-HLA class I (Biolegend), and anti-HLA-DR (Biolegend). The cells were analyzed in a flow cytometer (FACSCanto; BD Biosciences). Isotype matched control antibodies were used as controls. The T-MSCs used in this study expressed typical MSC markers.

1 × 10^6^ CD4^+^ T cells were stained with 1 µM CFSE and stimulated with none or 4 ug/mL PHA (Sigma-Aldrich, St. Louis, MO, USA) in the presence or absence of 5 × 10^4^, 1 × 10^5^, and 2 × 10^5^ T-MSCs. After 4 days, T-MSCs were harvested and analyzed by flow cytometry.

### 4.2. Assessment of Immunomodulatory Activity of Tonsil Derived Mesenchymal Stem Cell

To examine in vitro immunosuppressive activity of T-MSCs, we used tonsillar mononuclear cells (TMCs) as whole immune cells. Tonsil tissues were obtained by routine pediatric tonsillectomy. Those were cut manually into small pieces and exposed to enzymes, including collagenase type I and DNase I (Sigma-Aldrich) for 30 min at 37 °C under stirring. This solution was then filtered through a 70-µm cell strainer to collect single-cell suspensions. TMCs were obtained using Ficoll-Paque (GE Healthcare, Little Chalfont, UK) density gradient centrifugation. Whole immune cells (1 × 10^7^ cells in 1 mL of pre-warmed PBS) were labeled with CFSE (Invitrogen, St. Louis, MO, USA) at a final concentration of 5 µM. After incubation at 37 °C for 10 min, the cells were washed with complete medium containing RPMI 1640 (Hyclone, South Logan, UT, USA) supplemented with 10% FBS, 2 mM L-glutamine, 100 U/mL penicillin containing 10% FBS. The cells labeled with CFSE were cultured with 4 µg/mL of PHA and co-cultured with T-MSCs or primed T-MSCs with different concentration (5 × 10^4^, 1 × 10^5^, and 2 × 10^5^) for 4 days. After the co-culture, flow cytometric analysis of cell division by CFSE dilution was conducted to assess the proliferation of whole immune cells.

We also used CD4^+^ T cells to evaluate the immunosuppressive activity of T-MSCs. These were obtained from whole immune cells and purified using a CD4^+^ T cell Isolation Kit MicroBeads (Miltenyi Biotech, Bisley, Surrey, UK) according to the manufacturer’s instructions. For the T cell receptor (TCR) stimulation, purified CD4^+^ T cells were cultured in a complete medium containing RPMI 1640. In a 24-well plate, the wells were coated with 1 µg/mL anti-CD3 monoclonal antibodies (mAb; eBiosciences) at 4 °C overnight. The purified CD4^+^ cells were added to the wells at 1 × 10^6^ cells/mL and were stimulated with 3 µg/mL anti-CD28 mAb (eBiosciences) and 2 ng/mL IL-2 (eBiosciences) in 24-well culture plates. T-MSCs were co-cultured with CD4^+^ T cells at a ratio of 1:10 (T-MSCs: CD4^+^ T cells) for 3 days.

In this study, T-MSCs were primed with 10 ng/mL TNF-α and 20 ng/mL IFN-γ or treated PBS as vehicle control for 24 h. The primed T-MSCs (T-MSC^Primed^) or the control T-MSCs (T-MSC^Con^) were co-cultured with immune cells under PHA stimulation conditions. To prove IDO involvement, 1 mM of 1-MT as an inhibitor for IDO was added in culture media. Flow cytometric analysis was conducted to analyze the proliferation of immune cells and cytokine expression of CD4^+^ T cells. Moreover, to confirm the immunosuppressive properties of CD40 in the T-MSC^Primed^, purified CD4^+^ T cells were stimulated with anti-CD3 and anti-CD28 antibodies and co-cultured with T-MSC^Primed^ or T-MSC^Con^. Neutralizing anti-CD40 antibodies were added at a concentration of 1 µg/mL to the co-cultures of T-MSC^Primed^ and CD4^+^ T cells.

### 4.3. Flow Cytometry

To analyze the expression of cytokine of CD4^+^ T cells, cell surface staining was performed using pacific blue-conjugated CD4 (Biolegend) for 20 min at 4 °C in the dark. After the stained cells were washed with PBS, intracellular staining was performed to detect TNF-α, IFN-γ, IL-17A, and IL-4 expression. According to the manufacturer’s instructions, the cells were fixed and permeabilized using a Transcription Factor Staining Buffer Set kit (eBiosciences) and stained with allophycocyanin (APC)-cy7 conjugated TNF-α, (phycoerythrin) PE-cy7-conjugated IFN-γ, IL-4, or APC-conjugated IL-17A (eBiosciences). Prior to cytokine analysis, the purified CD4^+^ T cells were restimulated with 40 ng/mL phorbol-12-myristate-13-acetate (Sigma-Aldrich) and 1 µg/mL ionomycin (Sigma-Aldrich) for 5 h. Monensin (Sigma-Aldrich) was added at a concentration of 4 µM for the last 1 h of TCR stimulation or the last 6 h of PHA stimulation.

### 4.4. Gene Expression Analysis

Total RNA was extracted from T-MSCs using the easy-BLUE™ Total RNA extraction kit (Intron Biotechnology, Sungnam, Korea). cDNAs were synthesized from 2 µg total RNA using the Maxime RT PreMix kit (Intron Biotechnology) in a thermal cycler SimpliAmp™ Thermal cycler (Applied Biosystems, Foster City, CA, USA). Polymerase chain reaction (PCR) was performed with the following primers: indoleamine 2,3-dioxygenase (IDO)-1 (forward 5′-GCC CTT CAA GTG TTT CAC CAA-3′ and reverse 5′-GCC TTT CCA GCC AGA CAA ATA T-3′), COX-2 (forward 5′-GGT CTG GTG CCT GGT CTG AT-3′ and reverse 5′-TCC TGT TTA AGC ACA TCG CAT ACT-3′), heme oxygenase (HO-1) (forward 5′-ATG ACA CCA AGG ACC AGA GC-3′ and reverse 5′-GTG TAA GGA CCC ATC GGA GA-3′), TGF-β1 (forward 5′-GGG AAA TTG AGG GCT TTC G-3′ and reverse 5′ -GAA CCC GTT GAT GTC CAC TTG-3′), IL-10 (forward 5′-GGG AAA TTG AGG GCT TTC G-3′ and reverse 5′-GAA CCC GTT GAT GTC CAC TTG-3′), iNOS (forward 5′-GGT GGA AGC GGT AAC AAA GG-3′ and reverse 5′-TGC TTG GTG GCG AAG ATG A-3′), and beta actin (forward 5′-GTG CTA TCC CTG TAC GCC TC-3′ and reverse 5′-GGC CAT CTC TTG CTC GAA GT-3′). PCR was performed using a real-time thermal cycler system (Rotor GeneQ; Qiagen, Hilden, Germany).

### 4.5. Indoleamine 2,3-Dioxygenase Quantification

IDO production was measured by enzyme-linked immunosorbent assay (ELISA). According to the manufacturer’s instructions, the amounts of IDO were quantified using an ELISA kit (Solarbio Science and Technology, Beijing, China). The conditioned medium from primed T-MSCs with TNF-α and IFN-γ was collected, followed by measurement of the secreted IDO. Absorbance was measured on a microplate reader (Glomax; Promega, Korea).

### 4.6. Statistical Analysis

Experimental values are presented as the mean ± standard error of the mean. Statistical differences were determined by the Student’s t-test using the GraphPad Prism (version 5, Graph Pad Software, San Diego, CA, USA), and the statistical values were detailed in the figure legend.

## Data Availability

The authors confirm that the data supporting the findings of this study are available within the article.
